# DNA Metabarcoding Reveals Flying Arthropod Diversity and Community Turnover Across Ancient Tea Agroforestry Landscapes in Southwest China

**DOI:** 10.1002/ece3.74627

**Published:** 2026-11-02

**Authors:** Yanlan Xie, Yunyu Wang, Su Liu, Dan Wu, Feiyan Huang, Min He, Wanzhong Tan, Bingliang Xu, Hongrui Zhang, Yanhong Liu

**Affiliations:** ^1^ Yunnan Key Laboratory of Tea Germplasms Conservation and Utilization in the Lancang River Basin West Yunnan University Lincang Yunnan China; ^2^ Genome Center of Biodiversity, Kunming Institute of Zoology Chinese Academy of Sciences Kunming Yunnan China; ^3^ Yunnan Key Laboratory of Molecular Biology of Domestic Animals Chinese Academy of Sciences Kunming Yunnan China; ^4^ Plant Protection College Yunnan Agricultural University Kunming China

**Keywords:** agricultural heritage systems, agroforestry, beta diversity, community turnover, DNA metabarcoding, flying arthropods, forest connectivity, malaise trap

## Abstract

Ancient tea agroforestry landscapes in Southwest China are structurally complex systems that may support high arthropod diversity, yet their biodiversity value remains poorly documented. We used COI metabarcoding of Malaise‐trap bulk samples to assess flying arthropod assemblages at four tea‐associated sites in the Mengku Ancient Tea and Cultural System, Yunnan, China: a wild ancient tea tree community, a village‐adjacent cultivated ancient tea garden, a forest‐adjacent cultivated ancient tea garden, and a young‐shrub tea plantation. Samples were collected from May to October 2024 and analyzed using OTU‐based diversity and community‐composition approaches. We recovered 5669 arthropod OTUs. Diptera dominated the assemblages, accounting for 57.5% of total OTU richness, followed by Hymenoptera, Lepidoptera, Hemiptera, Coleoptera, and other arthropod groups. OTU richness, alpha diversity, and community composition varied among the four sites. WJC had the highest total OTU richness and mean alpha‐diversity values, whereas DXS yielded the most compositionally distinct assemblage and the largest number of OTUs detected exclusively at that site during this survey. Dominant families included natural enemies, herbivores, and mixed‐function taxa. Seasonal patterns showed higher OTU richness in early summer and September, but lower richness during the main rainy‐season months and October. These findings document marked spatial and seasonal variation in Malaise‐trap‐sampled flying arthropod assemblages. Because each landscape context was represented by a single site, the observed differences cannot be attributed independently to forest association, habitat heterogeneity, or management.

## Graphical abstract

We used COI metabarcoding of Malaise‐trap bulk samples to assess flying arthropod assemblages across four ancient tea agroforestry landscape contexts in the Mengku region of Yunnan, Southwest China. We recovered 5669 arthropod OTUs and found that forest‐associated and structurally complex tea landscapes supported high OTU richness, distinct community composition, and pronounced seasonal turnover, highlighting their value for biodiversity monitoring and conservation in traditional agroecosystems.

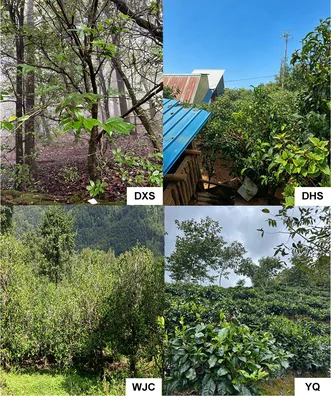

## 1 Introduction

Tea agroecosystems can support substantial biodiversity because perennial tea stands provide relatively continuous vegetation cover and can retain varying degrees of canopy, understory vegetation, and woody plant diversity. However, tea‐production landscapes differ markedly in vegetation structure, management history, surrounding land cover, and degree of human disturbance. These differences can influence habitat heterogeneity, microclimatic conditions, resource availability, and trophic interactions, with potential consequences for arthropod assemblages (Chowdhury et al. 2021). Understanding how arthropod communities vary among contrasting tea‐production contexts is therefore important for evaluating the biodiversity value of tea agroecosystems and for informing biodiversity‐sensitive management.

Previous studies have shown that arthropod diversity in tea and other perennial crop systems can differ substantially with habitat context and management. Beng et al. (2016), for example, reported declines in arthropod α‐diversity from forest to adjacent plantation habitats, although responses varied among taxonomic groups and crop types. Meng et al. (2021) found that forest‐associated tea systems supported greater diversity of total arthropods, herbivores, and natural enemies than monoculture tea plantations, whereas monocultures sometimes supported high arthropod abundance because of dominance by pest taxa. Local vegetation management can also modify arthropod assemblages: intercropping and ground‐cover vegetation have been associated with increased occurrence of predators and other natural enemies in tea plantations (Zhang et al. 2017; Chen et al. 2019). More broadly, retention of canopy and understory vegetation in agroforestry systems can increase habitat heterogeneity and provide resources and refuges for diverse arthropod functional groups (Klein et al. 2002; Altieri and Nicholls 2004).

Ancient tea agroforestry systems may be particularly important in this context because they combine long‐lived tea trees with heterogeneous woody vegetation and occur within landscapes that retain varying degrees of connection to natural forest. In southwestern China, the Shuangjiang Mengku Ancient Tea and Cultural System (SMATCS) contains a mosaic of wild ancient tea‐tree communities, traditionally managed ancient tea gardens, villages, natural forest, and more recently established tea plantations (Ma et al. 2017, 2020; Lei et al. 2022). Traditional ancient tea gardens can also provide habitat for non‐crop biodiversity. For example, Wu et al. (2024) showed that ancient tea gardens retaining shade trees and long‐term ecological continuity supported diverse epiphytic orchids and pollinator‐mediated reproduction. These findings suggest that ancient tea landscapes may function as important multifunctional habitats, but evidence for their arthropod biodiversity remains comparatively limited.

A key unresolved question is therefore not simply whether structurally complex or forest‐associated habitats contain more arthropods—a pattern broadly expected from agroecological theory—but how flying arthropod assemblages differ in richness, taxonomic composition, community turnover, and seasonal dynamics among the contrasting habitat and management contexts that coexist within an ancient tea cultural landscape. Existing tea‐agroecosystem studies have frequently focused on particular pest groups, natural enemies, or comparisons between conventional plantations and forest‐associated systems (Zhang et al. 2017; Chen et al. 2019; Meng et al. 2021). Much less is known about whole flying‐arthropod assemblages spanning wild ancient tea communities, traditionally cultivated ancient tea gardens, and recently established plantations within the same regional landscape. In particular, it remains unclear whether these contrasting contexts support largely overlapping assemblages or different subsets of regional arthropod diversity, and whether their temporal trajectories are similar or diverge through the growing season. Addressing these questions requires community‐level surveys capable of resolving large numbers of arthropod taxa across repeated sampling periods.

DNA metabarcoding of bulk Malaise‐trap samples provides an efficient approach for such community‐level characterization. Morphology‐based surveys of species‐rich arthropod samples are constrained by processing time, taxonomic expertise, and difficulties in identifying large numbers of small or damaged specimens. In contrast, DNA metabarcoding coupled with high‐throughput sequencing can recover large numbers of operational taxonomic units (OTUs) from mixed samples and has increasingly been applied to broad‐scale and repeated monitoring of flying arthropod assemblages (Piper et al. 2019; deWaard et al. 2019; Kirse et al. 2021; Wildermuth et al. 2023). Importantly, Malaise traps preferentially sample actively flying arthropods, and the resulting assemblages should therefore be interpreted as Malaise‐trap‐sampled flying arthropod communities rather than the entire arthropod fauna.

Here, we used Malaise‐trap sampling and COI DNA metabarcoding to characterize flying arthropod assemblages at four contrasting tea‐associated sites in Mengku Town, Shuangjiang County: a wild ancient tea‐tree community within forest habitat, a village‐adjacent cultivated ancient tea garden, a forest‐adjacent cultivated ancient tea garden, and a recently established young‐shrub tea plantation. These sites differ simultaneously in vegetation structure, forest association, elevation, tea age, management history, and local disturbance, and are therefore treated as contrasting landscape contexts rather than replicated levels of a single environmental gradient. Our study addressed three questions: (1) How do flying arthropod OTU richness and taxonomic composition vary among the four sampled sites? (2) To what extent do the sites share OTUs versus contain OTUs detected only within particular sites, and how strongly does overall community composition differ among them? (3) How do flying arthropod assemblages change from May to October, and do temporal patterns differ among the four sites? By addressing these questions, we aim to provide a community‐level baseline for flying arthropod biodiversity in an ancient tea agroforestry landscape and to identify patterns that can guide future replicated studies of the environmental and management factors structuring these assemblages.

## 2 Materials and Methods

### 2.1 Study Area and Sample Collection

The study area is located within the Shuangjiang Mengku Ancient Tea and Cultural System (SMATCS), situated in Mengku Town, Shuangjiang County, Lincang City, Yunnan Province, China (99°46′21″–99°58′27″ E, 23°33′–23°49′ N; Figure 1). The total area of the town is 475.3 km^2^, with elevations ranging from 1040 to 3233 m above sea level. The region experiences an annual average rainfall ranging from 1056 to 1800 mm, with a mean annual temperature of 22°C. The landscape is predominantly mountainous, with mountains covering 99.55% of the area and a forest cover rate of 72%. The region is characterized by a warm, humid climate and rich biodiversity (Ma et al. 2020). The total area of tea plantations in the region is 134.47 km^2^, with a particularly rich resource of ancient tea trees.

**FIGURE 1:**
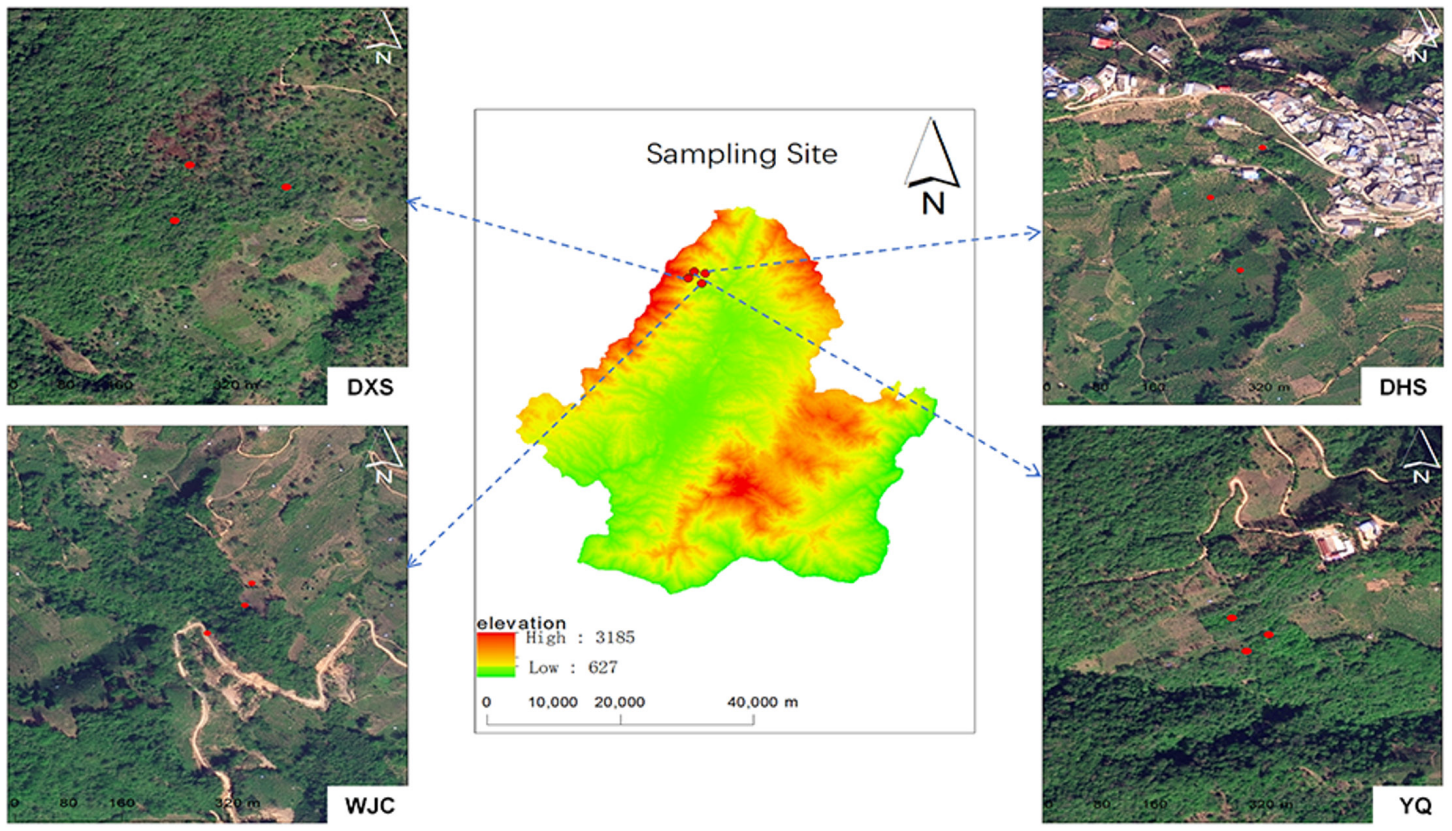
Location of the study area and four sampling sites in the Mengku ancient tea landscape. The central map shows the elevation gradient and location of the study area in Mengku Town, Shuangjiang County, Yunnan Province, China. The surrounding satellite images show DXS, a wild ancient tea tree community; DHS, a village‐adjacent cultivated ancient tea garden; WJC, a forest‐adjacent cultivated ancient tea garden; and YQ, a young‐shrub tea plantation. Red points indicate Malaise‐trap locations.

This study focused on four tea‐associated sites located along the mountain range west of the Nanmeng River in Mengku Town (Figure 1). The sites differed in forest association, village proximity, vegetation structure, elevation, tea‐garden age, and local management (Figure 2). For clarity, each site was assigned a site code based on its local site name. (1) Daxueshan (DXS) was a wild ancient tea tree community located in the forest interior of the Lancang River Provincial Nature Reserve at an elevation of approximately 2300 m a.s.l., more than 10 km from the nearest village. This site had minimal observed direct human disturbance. (2) Dahusai (DHS) was a cultivated ancient tea garden located adjacent to a village at an elevation of approximately 1900 m a.s.l., with most tea trees also over 100 years old. Owing to its close proximity to the settlement, free‐ranging poultry were frequently present within the tea garden. Tea trees were intercropped with *Juglans sigillata*, *Prunus persica* , *Castanea mollissima* , and *Ilex chinensis*, and some rows were interplanted with soybean, cabbage, and tomato. This site represented a village‐adjacent cultivated ancient tea garden with relatively higher human and livestock‐related disturbance. (3) Wujiacun (WJC) was a cultivated ancient tea garden adjacent to natural forest, located at an elevation of approximately 1600 m a.s.l. and more than 3 km from the nearest village. Most tea trees at this site were over 100 years old. The ancient tea trees were intercropped with *Ilex chinensis*, *Cinnamomum glanduliferum*, *Homalium ceylanicum*, *Cerasus serrulata*, and *Cunninghamia lanceolata* . This site represented a forest‐adjacent cultivated ancient tea garden with relatively low village‐related disturbance. (4) Yongquan (YQ) was a recently established small‐tree tea plantation located at an elevation of approximately 1900 m a.s.l., with tea trees approximately 15 years old. The plantation was established after the clearance of forest vegetation and is currently adjacent to natural forest, more than 5 km from the nearest village. Tea trees were intercropped with *Prunus persica* , *Prunus salicina* , and other native tree species. Compared with the ancient tea gardens, this site represented a younger and more recently converted cultivation type. No chemical inputs had been applied in any of the four tea gardens during the previous 3 years. Except for the wild ancient tea tree community, the other three tea gardens were harvested in spring and autumn, lightly pruned once in early June, and manually weeded two to three times per year.

**FIGURE 2:**
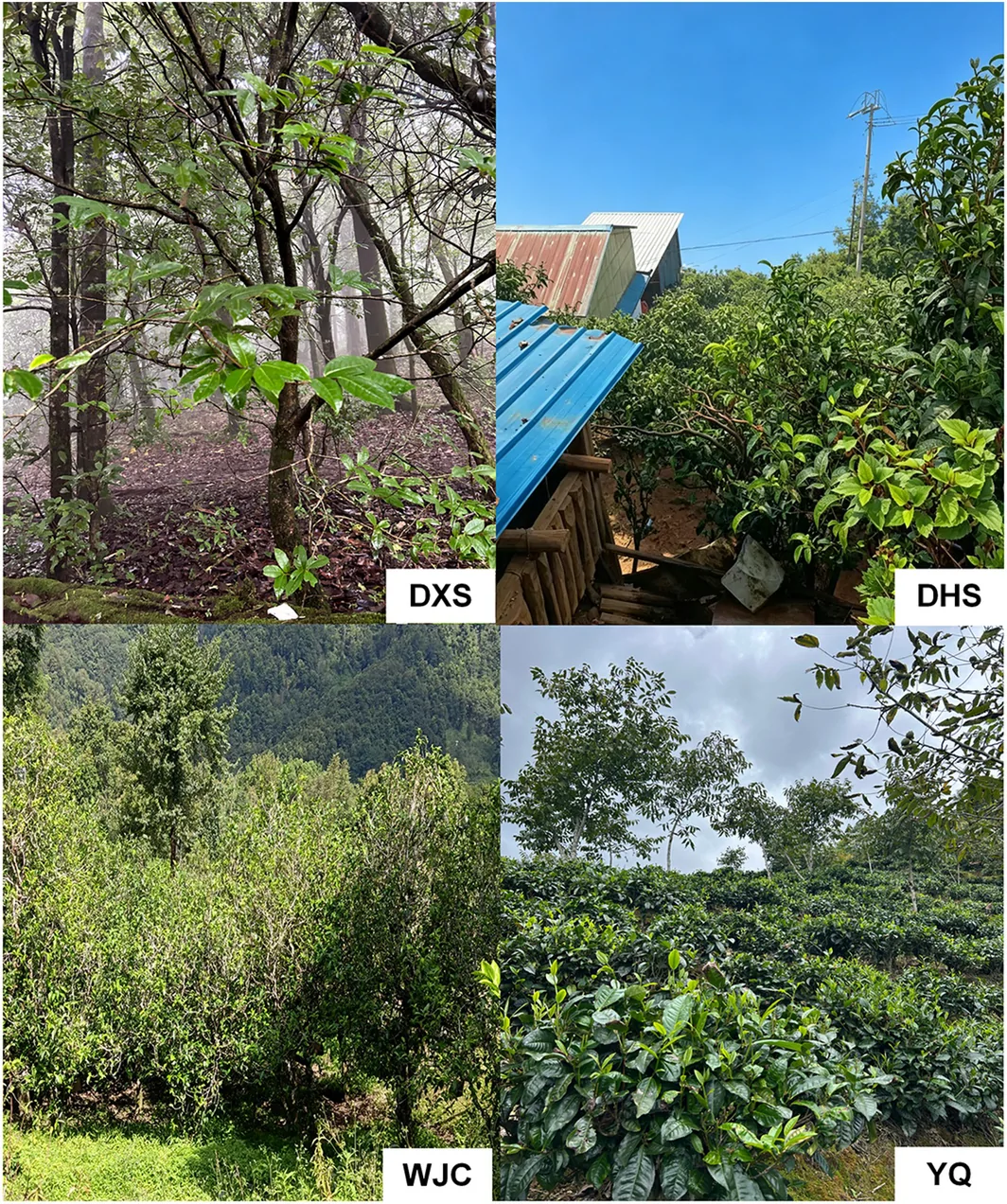
Habitat characteristics of the four sampled sites. Field photographs show DXS, DHS, WJC, and YQ and illustrate site‐level differences in vegetation structure, forest association, and management context. Photo credits: Yanlan Xie.

Within each tea garden, a 300 × 300 m plot was established. To minimize edge effects, all plots were located at least 100 m from habitat boundaries. Arthropods were collected using Malaise traps, with three traps placed at the northwest, central, and southeast positions within each plot, ensuring that the distance between any two traps exceeded 100 m. From May to October 2024, samples were collected biweekly from the Malaise traps, with collection bottles containing 400 mL of 95% ethanol. The collected samples were stored at −20°C. To optimize costs, only samples collected during the first 2 weeks of each month were subjected to DNA metabarcoding analysis, while the remaining samples were archived for future reference.

At each of the nine temporally matched sampling occasions selected for sequencing, catches from the three Malaise traps at each site were pooled before DNA extraction and processed as a single composite sample. The site‐by‐occasion composite therefore constituted the analytical unit, with the three traps serving as within‐site spatial subsamples rather than independent replicates. This design initially generated 36 composite libraries. Following bioinformatic filtering, one WJC library yielded no retained arthropod reads and was excluded, resulting in 35 libraries: eight complete four‐site sampling occasions and one occasion lacking WJC. Because each landscape context was represented by a single focal site, repeated sampling improved coverage of within‐season temporal variation but did not provide independent spatial replication of landscape context. Accordingly, the analyses account for sampling occasion and quantify differences among the four focal sites; they do not permit causal attribution of those differences to landscape context or management.

### 2.2 DNA Extraction and Metabarcoding

#### 2.2.1 DNA Extraction

The samples collected from the Malaise trap were in a mixed state, and DNA extraction was conducted using the “insect soup” method (Yu et al. 2012). Prior to extraction, samples were selected: larger insects (exceeding 5 mm in size) had a small piece of tissue removed, while smaller insects, such as mosquitoes, were used in their entirety for DNA extraction. All mixed insects were ground and digested in a 50 mL centrifuge tube after the alcohol had evaporated. The DNeasy Blood and Tissue Kit (Qiagen, Germany) was employed according to standard procedures. Notably, due to the large volume of mixed samples, we significantly increased the volumes of digestion buffer and Proteinase K (4500 μL ATL + 500 μL Proteinase K). Correspondingly, the volume of binding buffer was adjusted according to the manufacturer's guidelines, while the remaining steps strictly followed the outlined protocol. To ensure the reliability and accuracy of the results, a negative extraction control was included in the process.

#### 2.2.2 Metabarcoding

For DNA metabarcoding, we also employed the Leray–FolDegenRev primer set, which has been demonstrated to achieve high recovery of arthropod sequences from mixed DNA “soup” samples (Alberdi et al. 2018; Leray et al. 2013). The forward primer was mlCOIintF (Leray; 5′‐GGWACWGGWTGAACWGTWTAYCCYCC‐3′), and the reverse primer was Fol‐degen‐rev (5′‐TANACYTCNGGRTGNCCRAARAAYCA‐3′). We followed an optimized PCR protocol based on Yang et al. (2021), utilizing ‘twin‐tagging’ primers to allow detection and removal of tag‐jumped sequences, while adhering to the *Begum* strategy, which is an updated version of DAMe (Zepeda‐Mendoza et al. 2016) and available at https://github.com/shyamsg/Begum. Following the *Begum* strategy, each sample was PCR‐amplified three times, each time with a different‐tag sequence on a different plate. The same‐tag sequence was attached to the forward and reverse primers of a given PCR, which we call ‘twin‐tagging’, to allow detection and removal of tag‐jumped sequences, which produce non‐twinned tags. Each PCR experiment included both extraction and PCR negative controls. PCR setup was standardized with a 25 μL total volume, comprising 10 μL 2× PCRMIX premix, primers at 10 nmol/μL (1 μL each for forward and reverse), 2 μL eDNA template, and purified water to complete the volume. Cycling parameters included an initial denaturation at 95°C for 5 min, followed by 50 cycles of: 94°C for 30 s, 50°C for 30 s, and 72°C for 60 s.

#### 2.2.3 Illumina High‐Throughput Sequencing

The PCR products were combined in equal volumes, and PCR‐free sequencing libraries were constructed using the VAHTS Universal Pro DNA Library Prep Kit (Vazyme, ND608; 30–50 PCR products per library). Sequencing (2 × 150‐bp paired‐end) was performed on a NovaSeq 6000 platform (Illumina Inc., San Diego, California, USA). Library construction and sequencing were performed by Biolinker Technology Co. Ltd. (Kunming, Yunnan, China).

### 2.3 Data Processing

#### 2.3.1 Metabarcoding Bioinformatics Processing

Raw paired‐end reads were first quality‐checked and processed using *fastp* v0.23.4 (Chen 2023). Adapter sequences and low‐quality bases were removed, and paired‐end reads were merged using *fastp* with default settings, unless otherwise specified. The merged reads were subsequently processed using *Begum*, following the twin‐tagging strategy designed to identify and remove tag‐jumped and erroneous reads from replicated PCR libraries. Briefly, *Begum's sort.py* script was used to demultiplex sequences according to primer and tag combinations, append sample identifiers to sequence headers, and remove spacer, tag, and primer sequences. Reads carrying unexpected or non‐matching tag combinations were treated as putative tag‐jumping events and excluded from downstream analyses.

After demultiplexing, *Begum's filter.py* script was used to remove sequences outside the expected amplicon‐length range and to filter low‐frequency artifacts among the three independent PCR replicates. Following the replicate‐based filtering strategy of Yang et al. (2021), a sequence was retained only when it was detected in at least two of the three independent PCR replicates, with a minimum of four reads in each retained replicate. These criteria were applied within each biological sample before OTU clustering. Sequences failing either the replicate‐occurrence or minimum‐read criterion were excluded from downstream analyses. Chimeric sequences were removed using the de novo chimera‐detection algorithm in *vsearch v2.15.2* (Rognes et al. 2016). The remaining sequences were clustered into preliminary OTUs (Pre‐OTUs) at 97% sequence similarity using vsearch, yielding 9507 Pre‐OTUs. The OTU table was then curated using *LULU v0.1.0* (Frøslev et al. 2017), following metabarcoding workflows previously used by our research group (Wang et al. 2019, 2025). *LULU* identifies putative parent–daughter OTU relationships based on sequence similarity and nested co‐occurrence patterns. A minimum match similarity of 84% was used; this parameter was applied only during *LULU* curation and was not treated as a species‐delimitation threshold. After *LULU* curation, 8939 OTUs remained, corresponding to the consolidation of 568 Pre‐OTUs. OTUs represented by fewer than 10 reads across the entire dataset were subsequently removed as low‐frequency OTUs, leaving 5669 OTUs for downstream analyses.

Taxonomic assignments were performed by comparing OTU representative sequences against the NCBI nucleotide database using BLAST‐based searches implemented through the R packages *rentrez* (Winter 2017) and *Biostrings*. Assignments were evaluated based on sequence identity, alignment coverage, and consistency among the highest‐scoring matches. No fixed minimum alignment‐coverage threshold was applied; instead, coverage was considered together with sequence similarity and agreement among top matches. A sequence identity of ≥ 98% was treated as strong support for a potential species‐level assignment, but not as definitive evidence of species identity. OTUs lacking sufficient species‐level support were retained at the lowest reliable taxonomic rank, such as genus, family, or order. OTUs assigned to non‐target taxa, including bacteria, fungi, plants, other non‐arthropods, and Homo, were removed. OTUs detected in extraction or PCR negative controls were also excluded when they were considered contaminants or tag‐associated artifacts. All downstream diversity and community‐composition analyses were conducted at the OTU level, and richness is therefore reported as OTU richness rather than species richness.

#### 2.3.2 Statistical Analysis

Before statistical analysis, OTUs with zero counts across all retained libraries were removed. One composite library from WJC yielded no retained arthropod reads following bioinformatic filtering and was therefore excluded, leaving 35 libraries for analysis. For α‐diversity analysis, each retained library was rarefied to 161,951 reads, corresponding to the sequencing depth of the smallest retained library. Observed OTU richness, Shannon diversity, Simpson diversity (1 − *D*), and Pielou's evenness were calculated using the *vegan* package (version 2.6–8; Oksanen et al. 2024). Overall differences among sites were evaluated using the Skillings–Mack test, with sampling occasion treated as an incomplete block. This approach accommodated the single missing WJC library while retaining all 35 libraries in the analysis. For indices showing a significant overall difference, paired Wilcoxon signed‐rank tests were conducted for each site pair using only the sampling occasions shared by that pair. The resulting six pairwise *p* values were adjusted for multiple comparisons using the Holm procedure. All statistical tests were two‐sided, with *α* = 0.05.

For beta‐diversity analysis, non‐rarefied library counts were converted to within‐library relative sequence‐read abundances before Bray–Curtis dissimilarities were calculated. Community composition was visualized using principal coordinates analysis (PCoA), with Lingoes correction applied to address negative eigenvalues, and two‐dimensional non‐metric multidimensional scaling (NMDS) initiated from multiple random configurations to reduce the risk of convergence on a local minimum. Differences in community composition among sites were evaluated using a fixed‐block PERMANOVA in which sampling occasion was entered before site in the model, and 9999 permutations were restricted within sampling occasions. Thus, differences among sites were tested after accounting for temporally matched sampling. For each site pair, all admissible within‐occasion label permutations were enumerated across the sampling occasions shared by that pair, and the resulting pairwise *p* values were adjusted using the Holm procedure. Homogeneity of multivariate dispersion among sites was assessed using PERMDISP based on distances to the spatial median and the same restricted‐permutation structure. Ellipses shown in the ordination plots represent descriptive 95% normal‐theory data regions and should not be interpreted as confidence intervals for site centroids or as inferential tests of group separation. Taxonomic heat trees were generated using the *Metacoder* package (version 0.2.0; Foster et al. 2017) to visualize hierarchical taxonomic patterns in the taxonomic composition of the Malaise‐trap‐sampled flying‐arthropod assemblages.

Percentage stacked bar charts were used to display the relative contribution of arthropod orders to total OTU richness. A four‐way Venn diagram was generated using the *VennDiagram* package to show the numbers of shared and site‐specific OTUs among sampling sites. To evaluate the contribution of dominant families to community richness, the top 20 families ranked by overall OTU richness were selected, and the proportion of OTU richness contributed by these dominant families was calculated for each sample. These patterns were visualized using violin plots with overlaid jittered points, with red points indicating median values. Temporal dynamics of arthropod communities were visualized using the Biozeron Cloud Platform, which is based on CFViSA for visualization and statistical analysis of omics data (Wang et al. 2024).

Graphs were mainly generated using the *ggplot2* package (version 3.5.1) and the *ggpubr* package. Figure assembly was performed using the *patchwork* and *cowplot* packages, and axis labels were formatted using the *scales* package. Data processing was conducted using the *tidyverse* suite of packages (version 2.0.0). All statistical analyses were performed in R software (version 4.4.1; R Core Team 2024).

## 3 Results

### 3.1 OTU Detection and Flying Arthropod Community Composition

The rarefaction curves (Figure S1) indicate that the retained libraries approached sequencing plateaus, suggesting that the sequencing depth captured a substantial proportion of the detectable arthropod OTU richness across the sampled sites. Therefore, the observed differences among sites are less likely to have arisen solely from insufficient sequencing depth, although incomplete sampling cannot be entirely excluded.

DNA metabarcoding of Malaise trap samples successfully detected a total of 5669 OTUs across all four sampled sites from May to October 2024. The OTU distribution varied among sites, with WJC (forest‐adjacent ancient tea garden) showing the highest number of OTUs (2319), followed by YQ (young‐shrub plantation; 2172), DXS (wild ancient tea community; 2111), and DHS (village‐adjacent ancient tea garden; 1949) (Table 1). Overall, the four sampled sites yielded relatively high flying arthropod OTU richness, with clear differences among sites. Five major arthropod orders were detected. Diptera was dominant, comprising 3262 OTUs (57.5% of the total), followed by Hymenoptera (835 OTUs, 14.7%), Lepidoptera (654 OTUs, 11.5%), Hemiptera (428 OTUs, 7.6%), Coleoptera (319 OTUs, 5.6%), and other arthropod groups (171 OTUs, 3.0%) (Table 1). Diptera dominated all four sites, although the proportions of other orders varied among sites (Figure 3a). WJC had the highest OTU richness for Hemiptera, Lepidoptera, Coleoptera, and other groups, with 222, 267, 113, and 73 OTUs, respectively. Although DHS had the lowest total OTU richness, it contained the highest number of Hymenoptera OTUs (401 OTUs), accounting for 20.6% of the site‐level OTU richness.

**TABLE 1:** Number of OTUs detected by Malaise‐trap metabarcoding at each sampled site.

Order/Site	DXS	DHS	WJC	YQ	All
Coleoptera	93	103	113	94	319
Diptera	1393	1006	1368	1252	3262
Hemiptera	118	153	222	172	428
Hymenoptera	289	401	276	345	835
Lepidoptera	174	231	267	241	654
Others	44	55	73	68	171
All	2111	1949	2319	2172	5669

**FIGURE 3:**
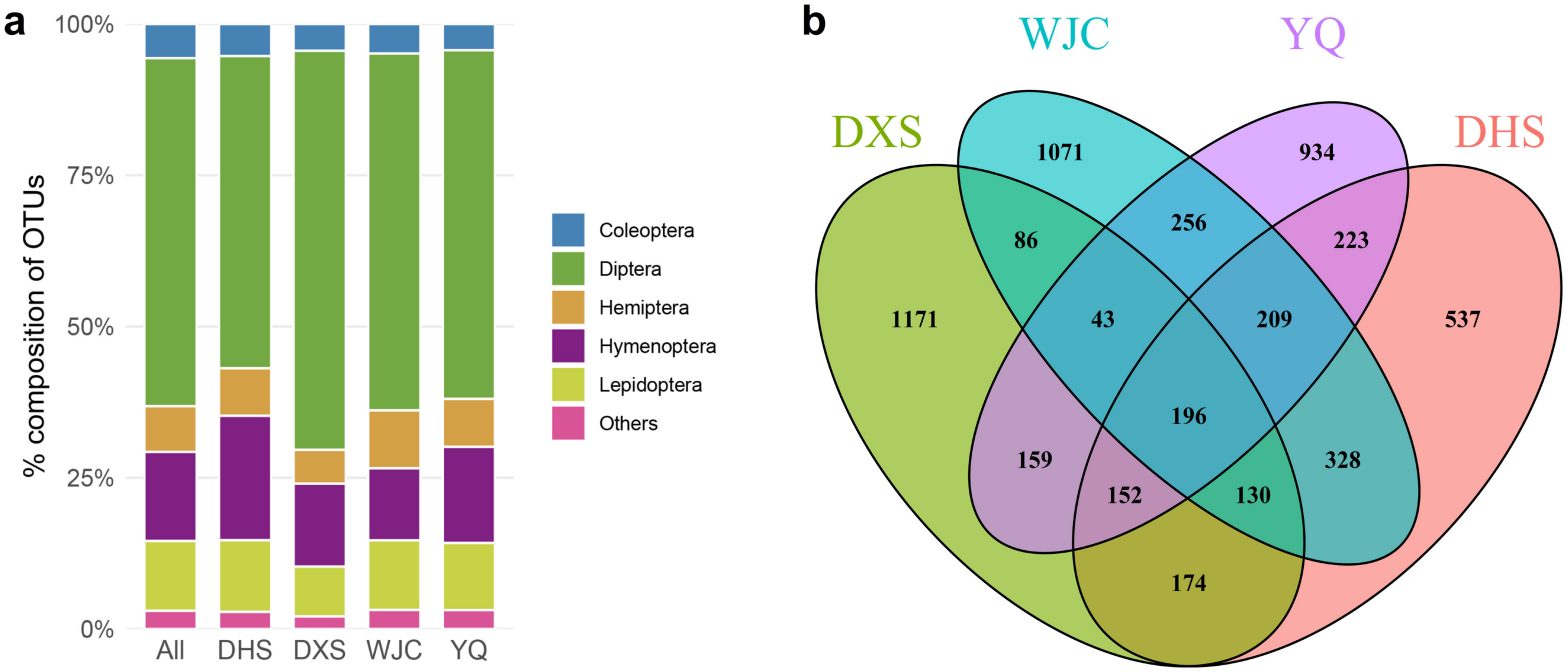
Malaise‐trap‐sampled flying arthropod community composition at the four sampled sites. (a) Relative composition of major arthropod orders based on OTU counts across DXS, DHS, WJC, and YQ. (b) Venn diagram showing OTUs shared among sites and OTUs detected at only one site during the survey.

The Venn diagram (Figure 3b) showed that only 196 OTUs were detected at all four sites, representing 3.5% of the total OTU pool. A further 3713 OTUs (65.5%) were detected at only one site during the survey. DXS harbored the highest number of unique OTUs, with 1171 OTUs, accounting for 55.5% of the OTUs detected at this site. This was followed by WJC with 1071 unique OTUs, YQ with 934 unique OTUs, and DHS with the fewest unique OTUs, at 537. These detection patterns indicate substantial turnover among sites, with DXS showing the largest site‐exclusive component. Pairwise comparisons showed that DHS and WJC shared 863 OTUs, DHS and YQ shared 780, and WJC and YQ shared 704, whereas DXS and WJC shared 455 OTUs. Thus, the three cultivated sites shared more OTUs with one another than either DHS or WJC shared with DXS. These presence‐absence summaries are descriptive; failure to detect an OTU does not establish its absence from a site.

### 3.2 Flying Arthropod Diversity Among the Four Sampled Sites

#### 3.2.1 Alpha Diversity

After exclusion of the empty WJC library and rarefaction of the remaining 35 libraries, WJC had the highest mean observed richness (625.75 ± 136.44), Shannon diversity (5.142 ± 0.386), Simpson diversity (0.981 ± 0.018), and Pielou's evenness (0.801 ± 0.043) (Table 2, Figure 4a–d). The Skillings–Mack test detected overall differences among sites in observed richness (SM = 9.181, *p* = 0.027) and Shannon diversity (SM = 8.271, *p* = 0.041), but not in Simpson diversity (SM = 6.738, *p* = 0.081) or Pielou's evenness (SM = 6.980, *p* = 0.073). No pairwise comparison remained significant after Holm adjustment.

**TABLE 2:** Alpha‐diversity indices of flying arthropod assemblages at the four sampled sites.

Diversity index/Site	DXS	DHS	WJC	YQ	Skillings–Mack *p*
Observed richness	494.22 ± 81.29	458.67 ± 96.89	625.75 ± 136.44	554.44 ± 129.15	0.027
Shannon index	4.551 ± 0.398	4.477 ± 0.659	5.142 ± 0.386	4.049 ± 1.122	0.041
Simpson index	0.963 ± 0.026	0.954 ± 0.041	0.981 ± 0.018	0.879 ± 0.125	0.081
Pielou's evenness	0.735 ± 0.053	0.731 ± 0.088	0.801 ± 0.043	0.640 ± 0.162	0.073

*Note:* Values are means ± standard deviations calculated from rarefied libraries. DXS, DHS, and YQ each had *n* = 9 libraries, whereas WJC had *n* = 8 after one empty library was excluded. *p* values are from Skillings–Mack tests with sampling occasion as the blocking factor. No pairwise comparison remained significant after Holm adjustment.

**FIGURE 4:**
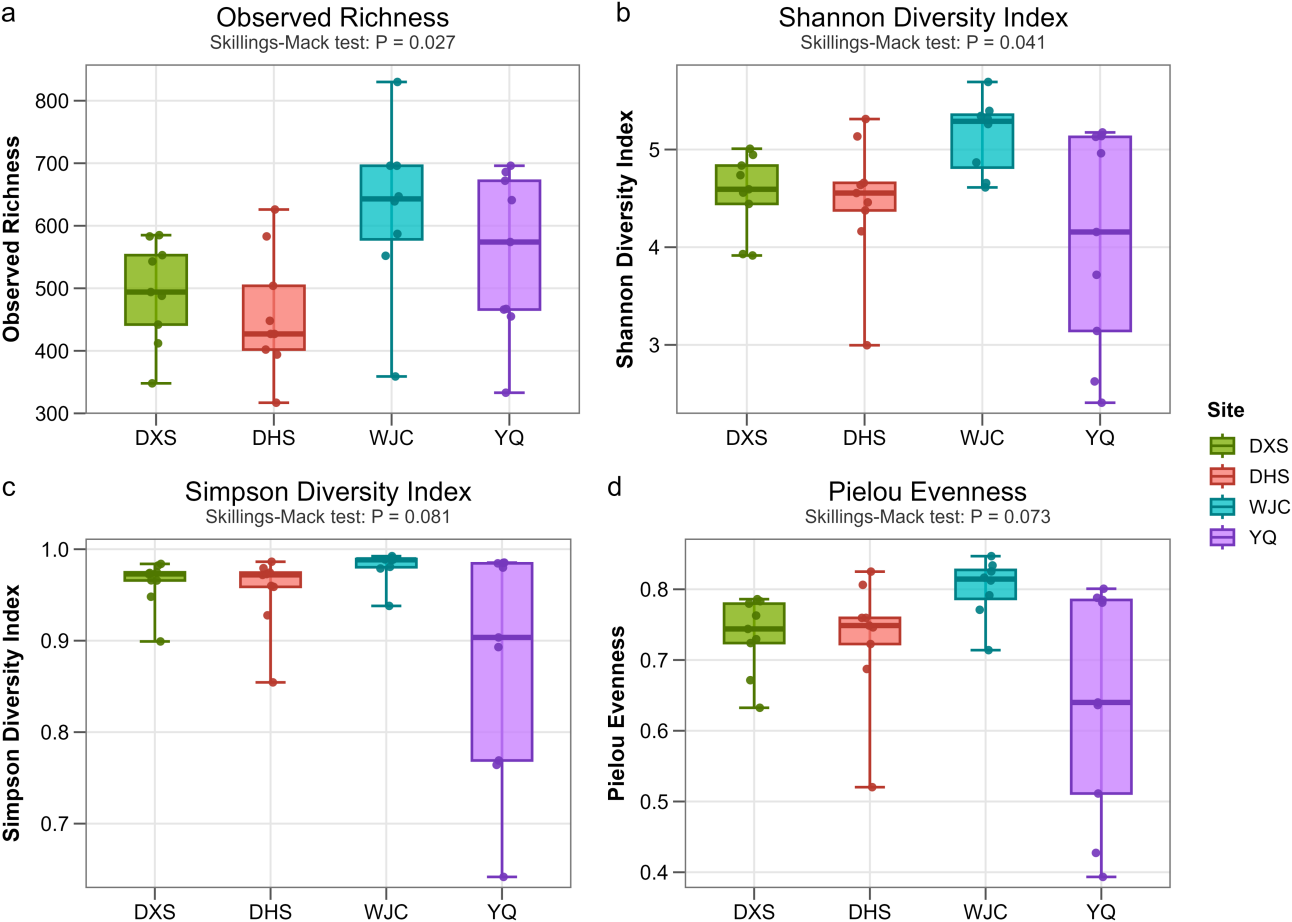
Alpha diversity of flying arthropod assemblages at the four sampled sites. (a) Observed OTU richness, (b) Shannon diversity, (c) Simpson diversity, and (d) Pielou's evenness. Points represent rarefied site‐by‐occasion composite libraries (DXS, DHS, and YQ: *N* = 9 each; WJC: *N* = 8). Panel annotations give overall Skillings–Mack *p* values with sampling occasion treated as an incomplete block; no pairwise comparison remained significant after Holm adjustment.

#### 3.2.2 Beta Diversity

Bray‐Curtis ordinations of the 35 retained libraries showed compositional differentiation among the four sampled sites (Figure 5). The first two axes of the Lingoes‐corrected PCoA explained 12.6% and 10.1% of the variation represented by the positive eigenvalues. The two‐dimensional NMDS solution converged after 68 random starts and had a stress of 0.156, supporting cautious interpretation of the two‐dimensional interpoint distances. After sampling occasion was included as a fixed block and permutations were restricted within occasions, the site term was significant (fixed‐block PERMANOVA: pseudo‐*F*(3, 23) = 4.373, *R* ^2^ = 0.283, *p* < 0.001). All six exact pairwise blocked comparisons remained significant after Holm correction (adjusted *p* = 0.023 for each comparison). PERMDISP provided no evidence of a difference in multivariate dispersion among sites (*F*(3, 31) = 0.997, *p* = 0.185).

**FIGURE 5:**
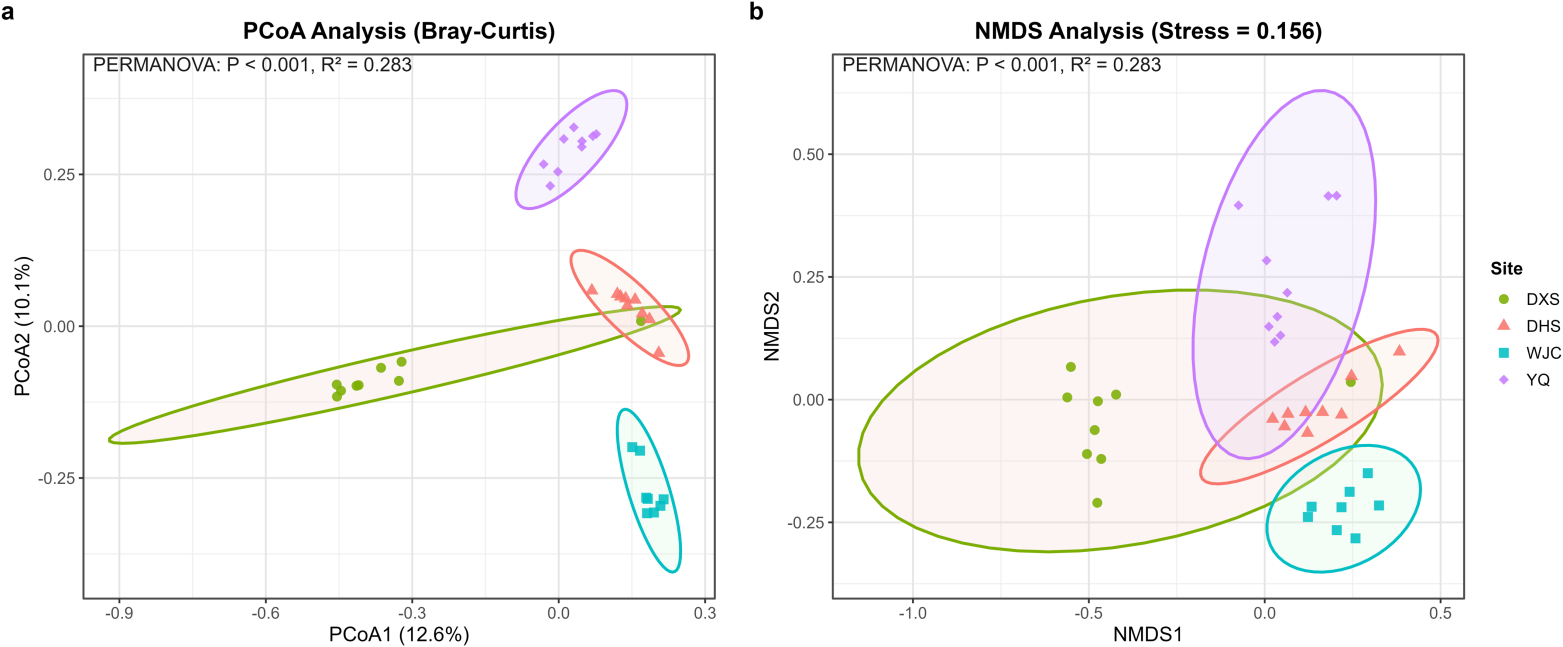
Bray‐Curtis ordination of Malaise‐trap‐sampled flying arthropod assemblages at four sampled sites. (a) Principal coordinates analysis with Lingoes correction and (b) two‐dimensional non‐metric multidimensional scaling. Points represent 35 site‐by‐occasion composite libraries. The first two PCoA axes explained 12.6% and 10.1% of the positive‐eigenvalue variation, and NMDS stress was 0.156. Ellipses are descriptive 95% normal‐data regions rather than confidence intervals for site means. Community composition differed among sites in the fixed‐block PERMANOVA (pseudo‐*F*(3, 23) = 4.373, *R* ^2^ = 0.283, *p* < 0.001), whereas multivariate dispersion did not (PERMDISP: *F*(3, 31) = 0.997, *p* = 0.185).

The taxonomic heat trees (Figure S2) provide a complementary descriptive summary of higher‐level taxonomic patterns among the four sites. Neither the heat‐tree comparisons nor the ordinations identify the environmental mechanisms underlying the observed differences. Because landscape context, elevation, vegetation structure, forest proximity, tea‐garden age, and management were not independently replicated, their individual contributions could not be separated.

### 3.3 Variation in Dominant Flying Arthropod Families Among Sampled Sites

The top 20 insect families collectively accounted for approximately 60% of total OTU richness across all samples, with Diptera contributing 10 of the 20 families (Figure 6a,b). Ichneumonidae (Hymenoptera), Phoridae, and Sciaridae (both Diptera) showed the highest proportions in individual samples, and some samples had more than 15% of their OTU richness assigned to a single family. Most dominant families varied considerably among samples, but no clear site‐level pattern was observed, suggesting that these families were broadly distributed across the four sampled sites.

**FIGURE 6:**
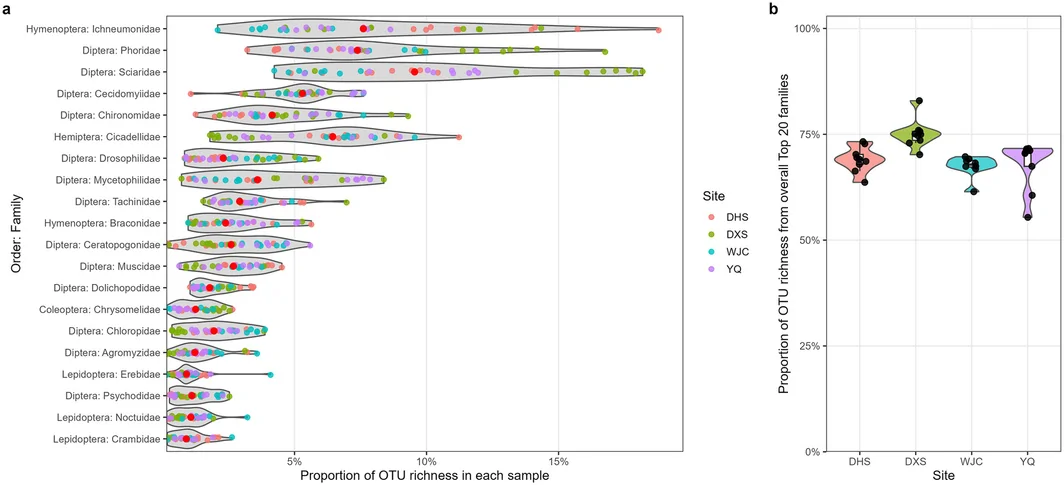
Violin plot analysis of the top 20 insect families at the four sampled sites. (a) Distribution of the relative contribution of each dominant family to OTU richness across individual samples. (b) Percentage of total insect OTU richness contributed by the top 20 families in each site. Points represent individual samples, and colors indicate different sampling sites: DHS, DXS, WJC, and YQ. Families are labeled as “Order: Family” and ranked according to their overall OTU richness across all samples.

Functionally, the top 20 families included pest or potential herbivorous insects, natural enemies, and neutral or mixed‐function insects. Pest‐related families were mainly represented by Cicadellidae, Chrysomelidae, Agromyzidae, Erebidae, Noctuidae, Crambidae, Cecidomyiidae, and Chloropidae, whereas natural enemies included Ichneumonidae, Braconidae, Tachinidae, and Dolichopodidae. Several dominant Dipteran families, including Phoridae, Sciaridae, Chironomidae, Drosophilidae, Mycetophilidae, Ceratopogonidae, Muscidae, and Psychodidae, were classified as neutral or mixed‐function groups. These results indicate that the dominant insect families were functionally diverse, rather than being composed only of pest‐related taxa.

### 3.4 Seasonal Dynamics of Flying Arthropod Assemblages

In addition to spatial differences, flying arthropod assemblages showed marked seasonal shifts from May to October 2024 (Figure 7 and Table S1). Across the four sampled sites, monthly OTU richness was generally higher in May–June and/or September, whereas a decline was observed during July–August in several sites, coinciding with the main rainy season in the region. OTU richness decreased markedly in October across most sites. The timing of peak OTU richness differed among sites: WJC showed relatively high OTU richness in May and September, YQ peaked in June, while DXS and DHS had relatively high richness in early summer, declined during the rainy‐season months, and showed a moderate rebound in September.

**FIGURE 7:**
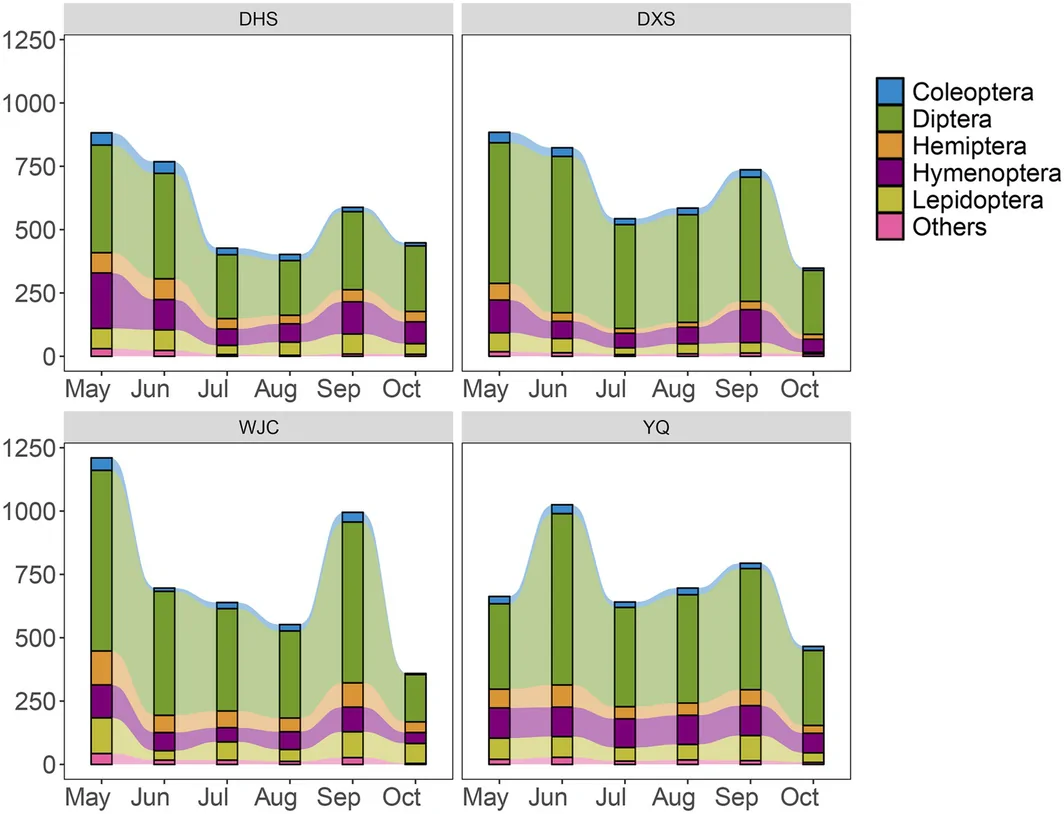
Seasonal dynamics of flying arthropod community composition at four sampled sites from May to October 2024. Panels show changes across sampling months (May–October) in order‐level OTU richness at each site.

Across all sites, Diptera contributed the largest number and proportion of detected OTUs throughout the sampling period, but its relative dominance weakened in late‐season months (especially September–October). Other orders showed stronger month‐to‐month variation in the numbers of detected OTUs. For example, Hemiptera and Hymenoptera exhibited noticeable temporal changes across the sampling period. Overall, seasonal turnover was evident in both order‐level OTU richness and community composition.

## 4 Discussion

This study used DNA metabarcoding to characterize the composition and seasonal dynamics of Malaise‐trap‐sampled flying arthropod assemblages at four tea‐associated sites in Southwest China. Diversity and community composition differed among sites and through time. The sites also differed in forest association, vegetation structure, elevation, tea‐garden age, and management, because these attributes covaried with site identity, their individual effects could not be separated.

### 4.1 Flying Arthropod Diversity and Community Differentiation Among Sampled Sites

The four sampled sites collectively yielded 5669 flying arthropod OTUs. WJC, the forest‐adjacent cultivated ancient tea garden, had the highest mean alpha‐diversity values and the greatest OTU richness for Hemiptera, Lepidoptera, and Coleoptera. This pattern is consistent with previous studies reporting higher arthropod diversity in forest‐associated and structurally complex production landscapes (Tscharntke et al. 2007, 2012; Meng et al. 2021), although forest proximity and vegetation structure were not tested independently here.

DHS, the village‐adjacent cultivated ancient tea garden, had the lowest overall OTU richness, despite harboring relatively high Hymenoptera OTU richness. The diverse intercropped plants and floral resources from agricultural plants such as soybean and cabbage may have contributed to the Hymenoptera pattern because these resources can attract parasitoid wasps and other flower‐visiting Hymenoptera (Klein et al. 2002; Altieri and Nicholls 2004). The lower overall richness at DHS may also be related to poultry activity, soil compaction, weeding, or intercrop management (Chowdhury et al. 2021), although these factors were not measured separately.

DXS had the largest number of OTUs detected exclusively at that site during this survey (1171; 55.5% of the OTUs detected at DXS), despite moderate total richness. Its assemblage also showed marked compositional differentiation from those at the other sampled sites, particularly WJC. This pattern is consistent with studies reporting distinct arthropod communities in forest interiors relative to agricultural habitats (Beng et al. 2016), but it cannot be attributed specifically to lower disturbance, closed‐canopy forest, or elevation in the present design.

YQ had relatively high observed OTU richness but lower Shannon, Simpson, and evenness values. Although many OTUs were detected, sequencing reads were unevenly distributed among OTUs. This pattern describes sequence‐read composition and should not be interpreted as evidence of organismal abundance or disturbance tolerance.

### 4.2 Functional Composition of Dominant Flying Arthropod Families

The top 20 insect families collectively accounted for approximately 60% of total OTU richness, a pattern similar to that reported in global analyses of flying insect diversity, in which a small number of families contribute disproportionately to local richness (Srivathsan et al. 2023). Diptera accounted for 10 of the top 20 families. This dominance may reflect both assemblage composition and the sampling characteristics of Malaise traps, which are particularly effective for flying insects. The results therefore describe the Malaise‐trap‐sampled flying arthropod assemblage; ground‐dwelling, weakly flying, and otherwise poorly intercepted arthropods are likely underrepresented.

The dominant families included taxa commonly associated with natural‐enemy functions (Ichneumonidae, Braconidae, Tachinidae, and Dolichopodidae), potential pest‐associated herbivores (Cicadellidae, Chrysomelidae, Agromyzidae, Cecidomyiidae, Erebidae, Noctuidae, and Crambidae), and neutral or ecologically mixed taxa (e.g., Phoridae, Sciaridae, and Chironomidae). These family‐level categories show broad functional representation in the catch but do not measure realized trophic interactions or ecosystem‐service rates. The relatively high OTU richness of parasitoid Hymenoptera, including Ichneumonidae and Braconidae, is consistent with their widespread occurrence in agroecosystems and their roles in biological pest regulation (Bianchi et al. 2006). Cicadellidae was also among the dominant families, consistent with previous reports from tea plantations (Meng et al. 2021).

The absence of a strong site‐level pattern among dominant families suggests that many of these families were detected across multiple sampled sites. Their relative contributions nevertheless varied among libraries, indicating fine‐scale heterogeneity in community structure. Microhabitat, vegetation composition, seasonal phenology, and local disturbance may contribute to this variation, but their effects could not be separated here.

### 4.3 Seasonal Dynamics and Temporal Turnover

Flying arthropod assemblages varied markedly from May to October. Monthly OTU richness was generally higher in May–June and September and lower during the mid‐rainy season (July–August) and in October. These patterns are consistent with the seasonal activity of subtropical montane insects in Yunnan (Chowdhury et al. 2021). Diptera contributed the largest number of OTUs throughout the survey, although its relative contribution declined late in the season, while Hemiptera and Hymenoptera varied more strongly among months. Plant‐resource availability and phenological synchrony may also contribute to these temporal patterns.

The timing of monthly OTU‐richness peaks also differed among sites. Descriptively, WJC and DXS showed less pronounced month‐to‐month fluctuations, whereas YQ showed greater within‐season variation.

### 4.4 Implications for Conservation and Sustainable Management of Ancient Tea Landscapes

The high OTU richness and marked compositional differences among the four sites document substantial local biodiversity within the sampled portion of the Mengku ancient tea landscape. No site ranked highest across all metrics: WJC had the highest mean alpha‐diversity values, whereas DXS had the largest number of OTUs detected only there during the survey. These complementary patterns suggest that maintaining heterogeneous tea‐associated habitats may help retain a broader range of flying‐arthropod assemblages at the landscape scale. However, because the four landscape contexts were not independently replicated, the site‐level patterns should not be generalized to landscape types. The findings are consistent with the view that traditional agroforestry systems can support biodiversity while maintaining agricultural production (Altieri and Nicholls 2004; Wu et al. 2024).

Broader agroecological evidence supports maintaining shade trees, forest edges, understory vegetation, intercropping systems, and habitat connectivity in tea‐producing landscapes. These features can provide resources and refuges for arthropods and natural enemies. Their effects in Mengku should be evaluated across independently replicated sites before specific management recommendations are made.

The relatively low richness at DHS identifies village‐adjacent management as a priority for further study. Free‐ranging poultry, weeding, soil disturbance, and vegetation simplification may influence flying arthropod assemblages, but these factors were not measured independently. Future work should compare replicated sites and quantify habitat and management variables directly.

## 5 Conclusions

This study provides a Malaise‐trap sampling and DNA metabarcoding‐based assessment of flying arthropod diversity and community composition at four tea‐associated sites in the Mengku region of Yunnan Province, China. A total of 5669 OTUs were detected across five major orders and other arthropod groups from May to October 2024.

WJC had the highest mean alpha‐diversity values, whereas DXS had the largest number of OTUs detected exclusively at one site during this survey. Observed richness and Shannon diversity differed overall among sites, although no pairwise alpha‐diversity comparison remained significant after Holm correction. Community composition differed among sites after sampling occasion was accounted for (fixed‐block PERMANOVA: pseudo‐*F*(3, 23) = 4.373, *R* ^2^ = 0.283, *p* < 0.001), and PERMDISP did not detect a significant difference in multivariate dispersion (*F*(3, 31) = 0.997, *p* = 0.185).

The top 20 insect families, dominated by Diptera, collectively accounted for approximately 60% of total OTU richness and included families associated with natural enemies, herbivores, and mixed ecological roles. OTU richness was generally higher in May–June and September and lower during the main rainy‐season months and October. Seasonal variation should therefore be considered when designing Malaise‐trap monitoring in the region.

Overall, this study documents differences in Malaise‐trap‐sampled flying arthropod diversity and community composition among four tea‐associated sites in the Mengku ancient tea landscape. The results provide a baseline for biodiversity monitoring but do not identify independent effects of forest proximity, vegetation structure, tea‐garden age, or management. Testing those relationships will require replicated sites and direct measurements of habitat, microclimatic, and management variables.

## Author Contributions

**Yanlan Xie:** conceptualization (lead), data curation (equal), formal analysis (equal), funding acquisition (equal), investigation (equal), methodology (equal), project administration (equal), software (equal), visualization (equal), writing – original draft (equal), writing – review and editing (equal). **Yunyu Wang:** conceptualization (equal), data curation (equal), formal analysis (equal), investigation (equal), methodology (equal), software (equal), visualization (equal), writing – original draft (equal), writing – review and editing (equal). **Su Liu:** data curation (equal), software (equal), visualization (equal), writing – review and editing (equal). **Dan Wu:** investigation (equal), writing – review and editing (equal). **Feiyan Huang:** investigation (equal), methodology (equal). **Min He:** investigation (equal), methodology (equal). **Bingliang Xu:** conceptualization (equal), methodology (equal), writing – review and editing (equal). **Wanzhong Tan:** conceptualization (equal), writing – review and editing (equal). **Hongrui Zhang:** conceptualization (equal), project administration (equal), supervision (equal), writing – review and editing (equal). **Yanhong Liu:** conceptualization (equal), funding acquisition (equal), project administration (equal), supervision (equal), writing – review and editing (equal).

## Funding

This work was supported by the Science and Technology Plan Project of Yunnan Provincial Department of Science and Technology (202401AT070167), the Key Laboratory Construction Project of Yunnan Provincial Department of Science and Technology (202449CE340010), and the Lincang “Xinglin Talent” Support Program (LCXLRC2024B—08).

## Conflicts of Interest

The authors declare no conflicts of interest.

## Supporting information


**Figure S1:** Rarefaction curves based on OTU richness versus number of reads. All samples show signs of reaching a sequencing plateau, indicating sufficient sequencing depth to capture most of the detectable arthropod diversity.
**Table S1:** Monthly OTU counts by order at each sampled site.
**Figure S2:** Metacoder taxonomic heat trees summarizing arthropod community composition at the four sampled sites (DXS, DHS, WJC, and YQ). Node sizes and colors represent relative taxonomic contributions as defined in the Metacoder output.

## Data Availability

The raw sequencing data have been deposited in NCBI under accession number PRJNA1473027.
