# Supplementary material for: DNA Metabarcoding Reveals Flying Arthropod Diversity and Community Turnover Across Ancient Tea Agroforestry Landscapes in Southwest China
Source: Ecol Evol. 2026 Nov 2;16(11):e74627. doi: 10.1002/ece3.74627 (PMC13728321; doi:10.1002/ece3.74627)
Supplement: Supplementary file 1 [file ECE3-16-e74627-s001.docx]

## Supporting information

FIGURE S1. Rarefaction curves based on OTU richness versus number of reads. All samples show signs of reaching a sequencing plateau, indicating sufficient sequencing depth to capture most of the detectable arthropod diversity.

**TABLE S1. Monthly OTU counts by order at each sampled site**

| **Site** | **Order** | **May** | **Jun** | **Jul** | **Aug** | **Sep** | **Oct** |
| --- | --- | --- | --- | --- | --- | --- | --- |
| DXS | Coleoptera | 41 | 34 | 23 | 26 | 29 | 9 |
|  | Diptera | 555 | 617 | 410 | 425 | 490 | 252 |
|  | Hemiptera | 66 | 34 | 19 | 19 | 33 | 20 |
|  | Hymenoptera | 129 | 68 | 58 | 66 | 130 | 51 |
|  | Lepidoptera | 75 | 56 | 26 | 39 | 41 | 7 |
|  | Others | 18 | 14 | 7 | 10 | 13 | 9 |
| DHS | Coleoptera | 48 | 46 | 26 | 24 | 17 | 12 |
|  | Diptera | 425 | 416 | 252 | 216 | 308 | 259 |
|  | Hemiptera | 80 | 82 | 41 | 34 | 48 | 41 |
|  | Hymenoptera | 219 | 120 | 65 | 72 | 127 | 86 |
|  | Lepidoptera | 80 | 81 | 36 | 52 | 79 | 42 |
|  | Others | 30 | 23 | 7 | 4 | 9 | 8 |
| WJC | Coleoptera | 49 | 13 | 24 | 25 | 38 | 4 |
|  | Diptera | 713 | 489 | 404 | 344 | 635 | 187 |
|  | Hemiptera | 134 | 68 | 66 | 54 | 96 | 42 |
|  | Hymenoptera | 130 | 72 | 56 | 70 | 97 | 43 |
|  | Lepidoptera | 141 | 37 | 72 | 47 | 102 | 79 |
|  | Others | 43 | 17 | 17 | 12 | 27 | 4 |
| YQ | Coleoptera | 29 | 35 | 21 | 26 | 21 | 16 |
|  | Diptera | 337 | 676 | 392 | 428 | 478 | 296 |
|  | Hemiptera | 74 | 88 | 48 | 48 | 63 | 31 |
|  | Hymenoptera | 119 | 116 | 113 | 115 | 118 | 77 |
|  | Lepidoptera | 84 | 82 | 54 | 61 | 99 | 38 |
|  | Others | 20 | 28 | 13 | 18 | 15 | 8 |

FIGURE S2. Metacoder taxonomic heat trees summarizing arthropod community composition at the four sampled sites (DXS, DHS, WJC, and YQ). Node sizes and colors represent relative taxonomic contributions as defined in the Metacoder output.
